# Improving the Alignment Quality of Consistency Based Aligners with an Evaluation Function Using Synonymous Protein Words

**DOI:** 10.1371/journal.pone.0027872

**Published:** 2011-12-02

**Authors:** Hsin-Nan Lin, Cédric Notredame, Jia-Ming Chang, Ting-Yi Sung, Wen-Lian Hsu

**Affiliations:** 1 Bioinformatics Lab, Institute of Information Science, Academia Sinica, Taipei, Taiwan; 2 Centre for Genomic Regulation (CRG), UPF, Barcelona, Spain; Russian Academy of Sciences, Institute for Biological Instrumentation, Russian Federation

## Abstract

Most sequence alignment tools can successfully align protein sequences with higher levels of sequence identity. The accuracy of corresponding structure alignment, however, decreases rapidly when considering distantly related sequences (<20% identity). In this range of identity, alignments optimized so as to maximize sequence similarity are often inaccurate from a structural point of view. Over the last two decades, most multiple protein aligners have been optimized for their capacity to reproduce structure-based alignments while using sequence information. Methods currently available differ essentially in the similarity measurement between aligned residues using substitution matrices, Fourier transform, sophisticated profile-profile functions, or consistency-based approaches, more recently.

In this paper, we present a flexible similarity measure for residue pairs to improve the quality of protein sequence alignment. Our approach, called SymAlign, relies on the identification of conserved words found across a sizeable fraction of the considered dataset, and supported by evolutionary analysis. These words are then used to define a position specific substitution matrix that better reflects the biological significance of local similarity. The experiment results show that the SymAlign scoring scheme can be incorporated within T-Coffee to improve sequence alignment accuracy. We also demonstrate that SymAlign is less sensitive to the presence of structurally non-similar proteins. In the analysis of the relationship between sequence identity and structure similarity, SymAlign can better differentiate structurally similar proteins from non- similar proteins.

We show that protein sequence alignments can be significantly improved using a similarity estimation based on weighted *n*-grams. In our analysis of the alignments thus produced, sequence conservation becomes a better indicator of structural similarity. SymAlign also provides alignment visualization that can display sub-optimal alignments on dot-matrices. The visualization makes it easy to identify well-supported alternative alignments that may not have been identified by dynamic programming. SymAlign is available at http://bio-cluster.iis.sinica.edu.tw/SymAlign/.

## Introduction

Experimentally determining a protein's structure is labor-intensive and time-consuming. Homology search, on the other hand, is a very effective way to predict the properties of an uncharacterized protein [1]. Homologous proteins tend to have similar structures. Their residue-residue equivalences can easily be established using any standard alignment procedure. This strategy is reasonably effective for proteins having more than 40% identity but its accuracy, defined as the capacity to identify structurally equivalent amino-acids, decreases significantly when considering remote homologues. It is quite well established that below 25% identity, sequence alignments become non-informative with respect to the structural similarity [2]. In this range of identity, an alignment based on amino-acid similarity may not be structurally correct. This situation is very common with remote homologues. In fact, the accurate alignment of remote homologues remains a major challenge for computational biology [3].

Most protein sequence alignment tools rely on a scoring function to measure the similarity between residues and give penalties for insertion/deletions. The quality of the resulting alignments is greatly influenced by the scoring function. The most common scoring functions are called substitution matrices. They include the PAM matrices [4], the BLOSUM series [5], GONNET [6], JTT [7], and VT [8]. Each substitution matrix is designed for a different purpose. For example, PAM matrices are designed to identify evolutionary origins of proteins, while the BLOSUM matrices are to identify protein members of the same family and to detect conserved domains. Selecting a substitution matrix is difficult as it is still not well understood which matrix is the best choice when aligning different sequence pairs. A recent study [9] concluded that the common belief that more accurate alignments of distantly related sequences may be achieved using low-identity matrices is shown to be false. Moreover, no evidence exists that selecting a matrix based on sequence divergence improves accuracy. This point is quite relevant, since the automated matrix selection is a key feature of ClustalW [10]. Substitution matrices are extremely simplified models of protein evolution. They assume all residues to evolve at the same pace, and ignore any local effect that may influence the mutability of a given amino-acid.

The last issue has been an intense focus of research over the last few years, and many methods have been produced that make an attempt to model amino acid mutations in a position specific manner. All these methods have their reliance on a pre-assembled multiple sequence alignment (MSA) used as a profile. They include Hidden Markov Models (HMMs) [11], [12], [13], [14], [15], [16], and Position Specific Scoring Matrices (PSSMs) which gives a weighted score or a probability reflecting the frequency of each residue at each position observed in a group of related sequences. PSI-BLAST [17] is probably the best known method to generate PSSM. Profile-sequence and profile-profile comparisons have been successfully applied on homology detection and fold recognition [12], [18], [19], [20]. A couple of multiple sequence aligners have also been developed that use the profile-profile alignment approach, which include PCMA [16], SATCHMO [21], [22], PRALINEpsi [23], SPEM [24], PROMALS [25] and PSI-Coffee [3]. The last three packages combine the profile-profile scoring scheme with a T-Coffee style consistency based scoring scheme. A study [26] found that profile-profile alignment gave an average improvement of 2–3% over profile-sequence alignment and ∼40% over sequence-sequence alignment. However, profile-profile alignment is not an entirely trivial process and the improvement depends heavily on the intrinsic properties of the considered dataset (average identity, density of the phylogenetic tree, etc). This probably explains why alternative studies [24] have found profile-profile alignment approaches to lack a clear superiority over traditional sequence-sequence alignment approaches.

Optimizing an MSA has been shown to be an *NP*-hard problem, hence a large number of methods have been developed to address this important biological problem [27]. A majority of the methods can be described as sophisticated variations around the progressive alignment algorithm, originally described by Hogeweg [28]. The algorithm starts with an all-against-all step where pairwise comparisons are carried out in order to fill up a distance matrix. This distance matrix is then resolved into a guide tree whose topology defines the order in which the sequences will be incorporated (one by one) within the MSA. This strategy is extremely time-effective, but it can suffer from its extreme greediness. Indeed, any pair of aligned sequences cannot be re-aligned, and early inaccurate alignments may impact the whole process through their influence on subsequent sub-alignments. The quality of an MSA is therefore very sensitive to the guide tree accuracy.

The excessive greediness of the progressive alignment can be tackled by improving the quality of the first pairwise alignments. This goal was the original motivation for the development of a novel class of progressive aligners known as consistency based progressive aligners. The original algorithm was developed for the T-Coffee and later re-implemented in several aligners including ProbCons [29] and PROMALS. The rationale of the consistency-based approach is to use the all-against-all computation in order to define a position specific scoring scheme for each pair of sequences that takes into account their relation with the other sequences. In this study, we decided to go further and extend the idea of the consistency based approach. Its principle relies on synonyms, a notion quite common in natural language processing. Its usage makes it possible to capture local sequence similarities. In this context, a protein synonym is an *n*-gram fragment of amino acids that reflects the sequence variation in the evolution. Synonymous *n*-grams can be effectively used to improve protein secondary structure predictions [30].

In this paper, we extend the application of synonyms to the problem of sequence alignment and present a method, called SymAlign, to demonstrate how protein synonyms can be used to improve the quality of protein sequence alignment. We applied this method to two well-known benchmark datasets and estimated the alignment quality using either reference alignments or structure based evaluation methods (RMSD and iRMSD). Our results show that SymAlign can generate alignments with better RMSD/iRMSD. We also demonstrate that SymAlign is less sensitive to structurally non-similar proteins when they are aligned together with structurally similar proteins. Finally, in the analysis of the relationship between sequence identity and structure similarity, we demonstrate that SymAlign can better differentiate structurally similar proteins from non-similar proteins.

## Methods

### The idea of protein synonyms

A synonym is a word that has identical or similar meaning as another word in the same language. For example, lovely, pretty, attractive, gorgeous, and so on all have similar meaning as beautiful but with different spelling in English. They are literally interchangeable with each other without changing the semantics of a sentence. Polysemy is the opposite phenomenon, that is to say the possibility for a single word to have different alternative meanings, depending on the context. For example, the word “play” could be used as a verb, in the sense “being involved in a game” or as a noun referring to a piece of writing performed by actors. The exact meaning of a polysemy depends on the context in which the word occurs. Technically, it is possible to align words in two sentences according to the meanings of known words and to infer the meaning of an unknown word from the word it is aligned with. We used this idea to propose the definition and identification of synonyms in protein sequences.

It is well known that a protein structure is encoded by its amino acid sequence. Therefore, a protein sequence can be treated as a text written in a language whose alphabet comprises 20 letters. The protein's structure is analogous to the semantic meaning of the text. However, the translation from sequence to structure remains a mystery. It is well known that homologous proteins share similar structures [31]. Therefore, we can learn something about protein language from those protein sequences.

We consider homologous proteins as sentences with similar meanings. They contain similar information and describe similar actions. Taking this analogy further, we may consider that the mutations between homologous sequences are like synonyms in two sentences. Thus we can create a mapping relationship between those sequence variations. In terms of natural language, a group of homologous sequences can be treated as a series of texts with identical or similar meanings. These texts can therefore be used to infer synonymous relationship from sequence alignments. The main limitation here is that we do not have much knowledge on how protein languages are composed and the precise rules or grammar indicating the boundaries of protein words.

Structure conformation is a highly complicated process where both long and short range interactions play an important role. Long-range interactions are very hard to model, but the short range ones can be effectively estimated using the *n*-gram model. The simplicity of the underlying modeling is very popular in formal linguistic analysis and bioinformatics sequence comparison. For instance, the BLAST's algorithm uses an *n*-gram model to generate a collection of similar words [32]. Our approach follows the same lines. Given a sequence alignment for proteins *A* and *B* with high sequence identity (typically above 30%), we use a sliding window to extract all word pairs to define synonyms. For each word *w* in protein *A*, if there is another word *w'* in protein *B* aligned with *w* and there is no gap between *w* and *w'*, we say *w* and *w'* are synonymous. The rationale is that since proteins *A* and *B* are similar in sequences, the two words would probably express similar structures. By extending the collection of proteins similar to protein *A*, we can define a larger number of synonyms for each word in *A*.

An important difference between protein synonyms and similar words in BLAST is that protein synonyms are defined from biologically significant sequence alignments (context-sensitive) while similar words are more or less context-free. Protein words share some properties with natural language words, including polysemy: the same protein word may appear in different proteins with different structures. Kabsch and Sander demonstrated that the same five residues can be a part of an alpha-helix in one protein and a part of a beta-strand in another protein, and they suggested that pentapeptide structure is strongly dependent on sequence context [33]. We can deal with this situation by restricting synonymous assignments to protein pairs similar enough.

**Figure 1:**
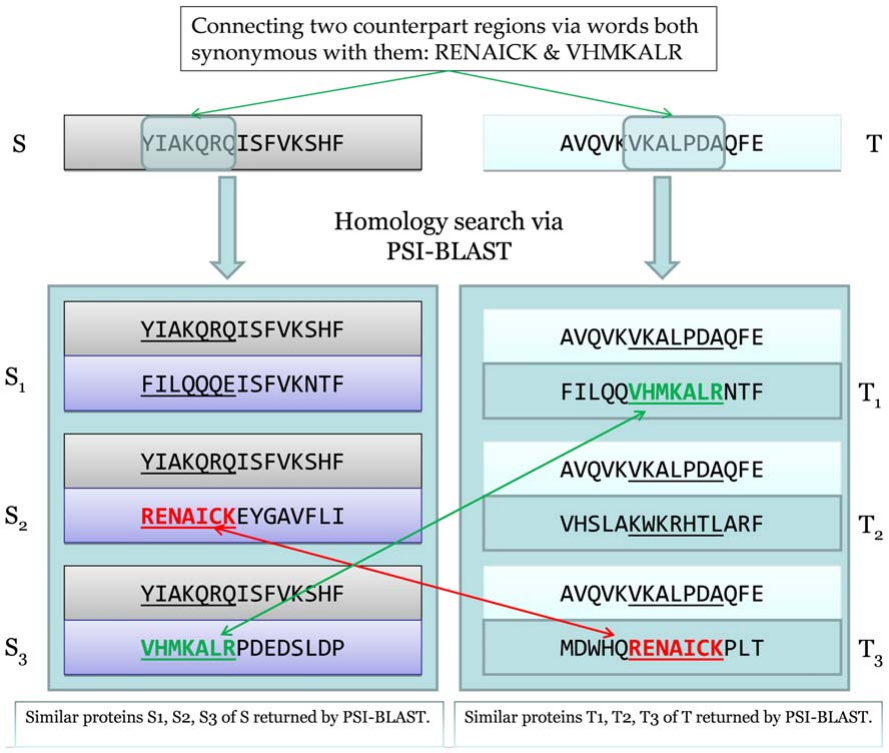
Connecting two counterpart regions by shared synonyms of two protein sequences. The words YIAKQRQ in protein *S* and VKALPDA in protein *T* share two synonyms which are extracted from their similar sequences.

In practice, given a target protein pair *S* and *T* to be aligned, we define synonyms for each word in *S* and *T* by using a sliding window to screen the sequence alignments between one of the target sequences and its similar sequences. We perform a PSI-BLAST search to generate a number of sequence alignments between *S* and *S'* (similar sequences of *S*). Each synonym *sw* extracted from *S'* is associated with a word in *S*. All synonyms for the words in protein *S* and *T* are collected this way. Whenever a word *sw* is found that are both synonymous with a word *w_s_* in *S* and a word *w_t_* in T, then *w_s_* and *w_t_* are considered to be synonymous by transitivity. Figure 1 shows how this idea can be used to connect regions of two protein sequences.

### The similarity measure of residue pairs

First, we introduce some notations used in this subsection. Consider we are given two sequences *S* and *T* to be aligned, *S* = *s_1_s_2_s_3_…s_m_* and *T* = *t_1_t_2_t_3_…t_n_*, where *s_i_*, *t_j_* are the *i*-th residue of *S* and *j*-*th* residue of *T*, respectively. A protein word *w_s_*_,*i*_ = *s_i_s_i_*_+1_ *…s_i+l_*_−1_ represents the subsequence of length *l*, which begins at *s_i_* and ends at *s_i+l_*_−1_. Let *sw_s_*_,*i*_ denote a synonym of the word *w_s_*_,*i*_, and F(*sw_s_*_,*i*_) be the frequency of *sw_s_*_,*i*_, which is the number of appearances among the similar proteins of *S*. Likewise, let *sw_t_*_,*j*_ represent a synonym of the word *w_t_*_,*j*_, and F(*sw_t_*_,*j*_) be the frequency of *sw_t_*_,*j*_. If *sw_s_*_,*i*_ = *sw_t_*_,*j*_, we say *w_s_*_,*i*_ and *w_t_*_,*j*_ share a synonym and we define a similarity score between *w_s_*_,*i*_ and *w_t_*_,*j*_ by *sim_s* = (F(*sw_s_*_,*i*_)+F(*sw_t_*_,*j*_))/2, which means that all of the residue pairs (*s_i_*, *t_j_*), (*s_i_*_+1_, *t_j_*_+1_), …, (*s_i_*_+*l*−1_, *t_j_*_+*l*−1_) are given a similarity score of *sim_s*. The final similarity score between a residue pair (*s_i_*, *t_j_*) is determined by the summation of *sim_s* for all common synonyms that cover residues *s_i_* and *t_j_*.

To avoid some residue pairs having much higher scores than others and resulting in a broken alignment, we normalize similarity scores by a simple normalization scheme. All the residue pairs are ranked by their similarity scores and we divide them into *N* groups equally, i.e., each group has equal number of residue pairs. The first group of residue pairs is assigned an alignment score of *N*, and the second group *N*−1, and so forth. *N* is set to be 500 in this study; however, if the number of alignable residue pairs is less than 500, then *N* is set to be the number of alignable residue pairs.

### Generating the alignment by T-Coffee

**Figure 2:**
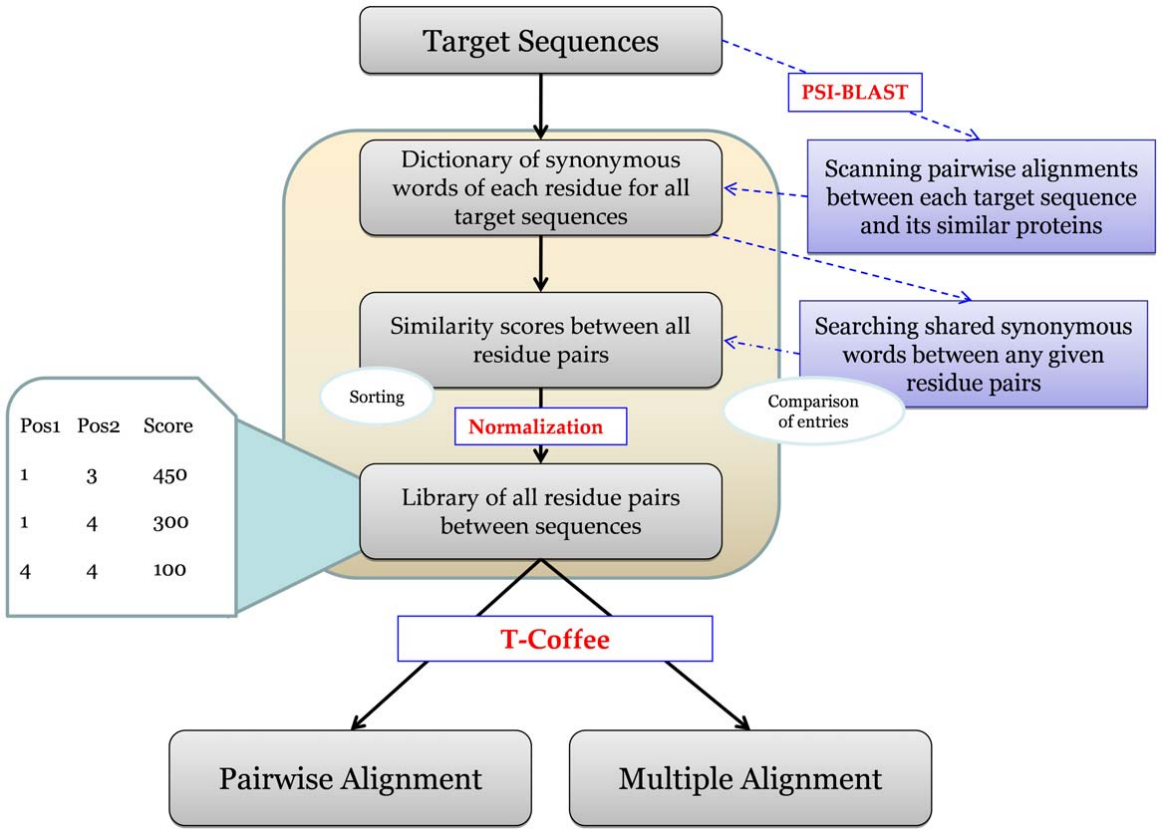
The algorithm of SymAlign. We use PSI-BLAST to collect a group of similar sequences for the targets from which we define synonyms. Similarity scores are estimated based on the shared synonyms. A library of all alignable residue pairs is made and fed into T-Coffee for generating a sequence alignment.

T-Coffee can generate an alignment based on customized alignment scores between residue pairs. We transform the similarity scores of all residue pairs into a library file. A library file is a list of pairs of residues that are considered alignable with a designated score. When dealing with MSAs, we generate a library file for each pair of protein sequences. T-Coffee builds a guide tree based on the input library files and reports a multiple sequence alignment. Figure 2 illustrates the complete algorithm of SymAlign.

### Two scoring functions for profile-profile alignment

In order to compare SymAlign with profile-profile alignment methods, we selected two scoring functions to calculate the alignment score for each residue pair and used T-Coffee to produce sequence alignments. The two scoring functions, *BASIC* [34] and *B-DHIP* [35], are described as follows.

BASIC is a profile–profile alignment method designed for fold recognition, which was successfully applied at CASP3 [36]. Given a pair of protein sequences *S* and *T*, BASIC is defined as follows:
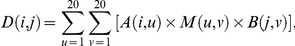
where *D*(*i*, *j*) is the alignment score for the residue pair (*s_i_*, *t_j_*), *A* and *B* represent the log-odds scoring matrices of the profiles for protein *S* and *T*, respectively, and *M* is the substitution matrix (*M* is BLOSUM62 in this study). BASIC compares two proteins from their profiles with the scores from the background comparison matrix.

B-DHIP is also a profile-profile alignment comparison method for fold recognition, which uses a standard dot-product between vectors of log-odds scores and probabilities of the 20 amino acids. It is defined as follows:
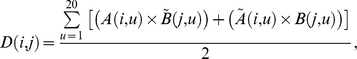
where *Ã* and *B* ~ represent the profiles of probabilities for the 20 amino acids at each position in *S* and *T*, respectively, while other symbols are same as those in BASIC. We generate all log-odds score and probability data for each protein sequence by PSI-BLAST.

### Performance evaluation

To estimate the accuracy of a sequence alignment, researchers often use a reference-dependent measure, the quality score (Q-score). It is defined as the ratio of the number of correctly aligned residue pairs compared with the reference alignment. The measure that depends on a reference alignment has been criticized for the limited capacity to capture the structural correctness of an alignment globally [37]. An alternative is to compute a reference-independant measure, for instance, by estimating the quality of the superposition induced by the alignment. Two such metrics have been proposed: the RMSD (Root Mean Square Deviation) that estimates the quality of the superposition, and the iRMSD (intra molecular Root Mean Square Deviation) [37] that estimates the difference of intra-molecular distances between pairs of equivalent alpha carbons in a way similar to APDB [38].

In this study, we evaluated the performance using Q-score, iRMSD and RMSD. We downloaded and modified the program from the http://boscoh.com/protein/rmsd-root-mean-square-deviation, which is used to calculate the RMSD between PDB structures based on Kabsch's algorithm [39]. To simplify the calculation of the RMSD, each amino acid is represented by the 3D coordinates of the nitrogen (*N*), α-carbon (*CA*), carbon (*C*) and oxygen (*O*) atoms on its backbone. Because RMSD is used to measure the difference between two protein structures, we only evaluated the performance of pairwise sequence alignment. When measuring the performance of multiple sequence alignments, we calculated the average RMSD between every pair of structures within the MSA. The iRMSD was estimated using the implementation distributed with the T-Coffee package.

### Benchmark datasets

We assessed the performances on two benchmark databases: BAliBASE 3.0 [40] and PREFAB 4.0 [41]. BAliBASE consists of eight reference sets, meant to reflect real alignment problems. Since most of the existing sequence alignment tools can successfully align sequences sharing >40% identity but fail for more divergent sequences with <20% identity, we focused on those divergent sequences in this study, And this subset, referred to as RV11, has been shown to be most informative and predictive of the ranking [3]. Furthermore, it is one of the few datasets in BAliBASE where all aligned members have a known structure, which serves to minimize the methodological biases [41]. RV11 consists of 38 test sets. Since BB11037 is the only test set that does not contain PDB structures, it was excluded from this study. The remaining RV11 contains a total of 256 sequences, distributing exclusively in 37 MSAs and making up 933 pairwise alignments with an average identity of 11.54%. BAliBASE alignments can be evaluated either on the entire alignments or on pre-defined blocks (core regions). The definition of these core regions is somehow arbitrary and has recently been criticized. For this reason, we decided to do the analysis on the entire sequences.

PREFAB 4.0 contains 1,682 pairwise reference alignments. We filtered out some proteins that do not have PDB structures or whose sequences are inconsistent with the associated PDB entries. We also filtered out protein pairs having more than 20% identity. The resulting subset includes 1,553 sequences and 954 pairwise alignments. The average sequence identity is about 10.01%. Like RV11, we conducted the analysis on the entire sequences in PREFAB and ignored the annotation of core regions.

### Sequence Alignment Packages

We compared the performance of SymAlign with eight state-of-the-art methods: ClustalW (version 2.1) [10], Dialign (version 2.2.2) [42], MAFFT (version 6.847beta) [43], MTRAP (version 1.2) [44], MUSCLE (version 3.8.31) [45], Probalign (version 1.4) [46], T-Coffee (version 8.97_101117) [47], and ProbCons (version 1.12) [29], as well as the two scoring functions for profile-profile comparison BASIC and B-DHIP described in this section.

## Results and Discussion

### Comparison with existing methods on pairwise alignments

The comparison of pairwise sequence alignments on the BAliBASE and PREFAB are summarized in Table 1. It is worth noting that for the reference-independant measures, the best scores are estimated on the reference alignments: 1.28 Å and 6.81 Å for the iRMSD and the RMSD on BAliBASE's RV11, 1.15 Å and 6.25 Å on PREFAB. The consistency-based methods outperform the others in terms of Q-score, RMSD, and iRMSD. The results of most methods on the two datasets are in reasonable agreement in the three metrics, and with slightly more fluctuations when considering consistency-based methods.

**Table 1:** Comparison with existing methods on pairwise alignments.

Methods	BAliBASE's RV11			PREFAB		
	Q-score (%)	iRMSD (Å)	RMSD(Å)	Q-score (%)	iRMSD (Å)	RMSD(Å)
SymAlign	45.78	1.31	11.10	22.56	1.35	11.57
Probalign	40.42	1.38	11.84	21.10	1.40	11.89
MTRAP	39.71	1.41	12.97	21.80	1.44	12.91
T-Coffee	39.58	1.38	13.10	21.45	1.43	13.30
ProbCons	38.76	1.38	13.15	21.03	1.40	13.23
MUSCLE	37.44	1.42	13.23	21.18	1.47	13.29
MAFFT	35.35	1.41	13.63	19.13	1.45	13.78
ClustalW	34.21	1.48	13.69	19.14	1.50	13.29
B_DHIP	27.44	1.48	13.75	13.81	1.43	12.93
Dialign	29.78	1.42	14.91	15.71	1.42	14.00
BASIC	14.91	1.41	16.73	8.57	1.48	14.86

Every pair of proteins contained in each test set was aligned with each aligner and subsequently evaluated with the three metrics: Q-score, iRMSD and RMSD. SymAlign achieves the best ranking on the two test sets and the three quality measures.

SymAlign achieves the best performance on the two datasets in terms of all the three measures, and Probalign achieves the second best ranking in iRMSD and RMSD measures. Since SymAlign computes the similarity scores of residue pairs and uses T-Coffee to generate alignments, the results of the two methods can be directly compared. SymAlign achieves an improvement of 6.2% in Q-score and 2 Å in RMSD over T-Coffee on BAliBASE's RV11, and 1.11% in Q-score and 1.73 Å in RMSD on PREFAB.

Furthermore, observed from Table 1, it is noteworthy that Probalign, MTRAP, T-Coffee, ProbCons and MUSCLE achieve equal performance in Q-score (ranging from 21.03% to 21.80% on PREFAB), though their average RMSDs vary from 11.89 Å to 13.30 Å. It shows that RMSD can better demonstrate the alignment quality in terms of the resulting structural superposition. However, the differences in iRMSD among these methods are not distinguishable. It suggests that RMSD is a better choice to estimate the alignment quality than iRMSD. It is also interesting to note that, the scoring functions of profile-profile comparisons do not perform better than traditional sequence-sequence comparison methods.

### Comparison with existing methods on multiple alignments of the benchmark datasets with and without outliers

We further compared these methods on multiple sequence alignments of the BAliBASE's RV11, and the results are shown in Table 2. Probalign achieves the best RMSD on RV11. SymAlign achieves the second best, showing a 1.11 Å improvement over T-Coffee. It can also be observed that the RMSDs of all of methods get improved compared with results on pairwise alignments except MTRAP. In general, multiple alignments can achieve better alignment quality since they can benefit from pairwise alignments through either the progressive alignment strategy, or via the consistency-based scheme.

**Table 2:** Comparison with existing methods on multiple alignments and outliers.

Methods	1. BAliBASE'sRV11	2. BAliBASE'sRV11' (with outliers)
	RMSD(Å)	RMSD (Å)
SymAlign	9.20	9.40
Probalign	8.70	10.20
T-Coffee	10.31	10.80
ProbCons	10.31	11.09
MUSCLE	11.75	13.39
Dialign	11.90	11.64
MAFFT	12.21	13.89
ClustalW	12.44	13.39
MTRAP	16.38	16.53

We estimated the alignment accuracy on the original RV11 test sets and those with additions of outliers. The experiment result shows that SymAlign is more robust to outliers than any other aligners tested here.

Note that the protein members in each MSA of RV11 are specially selected. Although their mutual sequence identities are below 20%, they are structurally similar. This defines a very challenging situation albeit not entirely realistic. In practice, it is common to align a set of sequences containing non-homologous ones. Unfortunately, the progressive alignment procedure can be strongly affected by non-homologous sequences. In order to estimate the alignment quality when facing such problem, we simulated this situation by adding “outliers” into each test set of RV11 as described below. Given two sequences *P_i_* and *P_i_*′, let *SeqIdy*(*P_i_*, *P_i_*′) denote the sequence identity of *P_i_* and *P_i_*′ calculated by T-Coffee, and *TMscore*(*P_i_*, *P_i_*′) denote the TM-score of *P_i_* and *P_i_*′. Note that TM-score is a score between (0,1] to measure the similarity of topologies of two structures [48], which is estimated by TM-align [49], a structural alignment program. The higher the score, the more similar the two structures. Given a test set *U* = {*P_1_*, *P_2_*, …, *P_k_*} of RV11, we generated an outlier *P_i_*′ for each *P_i_*, where *P_i_*′ is selected from any other test set that maximizes the difference between *SeqIdy*(*P_i_*, *P_i_*′) and *TMscore*(*P_i_*, *P_i_*′). We then tested on the new test set *U*' given by {*P_1_*, *P*_2_, …, *P_k_*, *P*_1_′, *P*_2_′, …, *P_k_*′} For example, we add proteins *BB11035.1dvh*, *BB11002.1ihv_A*, *BB11033.1erv*, and *BB11026.1ubi* into *BB11001* as outliers. The new test set *BB11001'* includes four original protein members and the four outliers.

We intended to disturb the guide tree by adding outliers. The outliers are involved in the process of MSA, but not involved in the evaluation of alignment quality. They serve as noises to test the robustness of an alignment tool. To compare the performance of different tools on the new dataset RV11 with outliers (denoted as RV11'), we calculated the RMSD on the original protein pairs according to the resulting MSA. The comparison results on RV11' are shown in the second column of Table 2. It can be observed that SymAlign achieves the best performance, which suggests that SymAlign is robust to outliers while Probalign, MUSCLE, and MAFFT are more sensitive to outliers, and their RMSDs are increased by more than 1.5 Å.

### Identification of structural similarity

The performance of a method on benchmark datasets may not always be representative of its performance on real datasets. Indeed, a real dataset sometimes includes protein sequences that may not be structurally similar. However, there is little discussion in the literature about the alignment quality for protein sequences with no structural similarities. In reality, given sequence alignments with <20% identity, one has difficulty in distinguishing between structural similarity and non-similarity no matter how accurate an aligner can achieve on these sequences. Thus in this section, we evaluated each method by how its alignment results reflect structural similarity or non-similarity.

To this end, we generated all possible sequence pairs in BAliBASE's RV11 and PREFAB, i.e., 31,707 and 1,205,128 pairs, respectively. A recent study on TM-align [50] suggested that protein pairs with a TM-score >0.5 are mostly in the same fold while those with a TM-score <0.5 are mainly not in the same fold. We used TM-Align to label each pair as a proven positive (PP) if TM-score ≥0.5 or a proven negative (PN) if TM-score <0.5. In BAliBASE's RV11, 1.13% of the pairs were labeled as PPs and 1.09% in PREFAB. We aligned all of the pairs by each method considered here and evaluated the usefulness of sequence identities to indicate correct PP or PN labels. We used thee different thresholds of sequence identity, i.e., >10%, >15%, and >20%, to predict each sequence pair to be structurally similar. We then calculated the precision = TP/(TP+FP) and the recall = TP/(TP+FN). The results on RV11 and PREFAB are summarized in Tables 3 and 4, respectively.

**Table 3:** The comparison results of identifying structural similarity on RV11.

Method	Sequence Identity >10%		Sequence Identity >15%		Sequence Identity >20%	
	Precision	Recall	Precision	Recall	Precision	Recall
TM-align	93.40	23.74	100.00	10.61	100.00	6.42
SymAlign	40.57	23.46	81.13	12.01	100.00	7.26
Dialign	6.69	28.21	28.77	11.17	90.00	7.54
ClustalW	1.65	65.36	18.46	20.11	85.71	8.37
MTRAP	1.63	65.08	17.41	21.78	88.89	8.94
Probalign	1.61	63.13	6.62	26.53	85.41	11.45
T-Coffee	1.55	74.02	6.44	28.49	77.77	9.77
ProbCons	1.52	74.86	5.96	32.12	74.00	10.33
MUSCLE	1.47	83.80	3.39	38.26	72.73	11.17
MAFFT	1.32	88.83	1.66	60.05	19.87	17.32

**Table 4:** The comparison results of identifying structural similarity on PREFAB.

Method	Sequence Identity >10%		Sequence Identity >15%		Sequence Identity >20%	
	Precision	Recall	Precision	Recall	Precision	Recall
TM-align	94.53	14.55	98.42	7.59	98.75	4.22
SymAlign	14.25	15.68	70.41	8.70	95.89	4.78
Dialign	3.03	24.77	22.65	9.55	87.14	4.88
MTRAP	1.42	50.71	10.68	14.38	87.02	5.74
Probalign	1.41	51.29	5.07	19.15	70.44	6.79
ClustalW	1.39	52.46	9.26	14.56	81.08	5.79
T-Coffee	1.27	58.22	4.34	20.52	59.45	6.91
ProbCons	1.25	60.52	3.84	21.59	55.05	7.18
MUSCLE	1.17	67.25	2.82	25.87	53.63	7.16
MAFFT	1.10	79.29	1.48	45.02	13.69	10.92

SymAlign consistently achieved the highest precisions among all sequence aligners at the three different identity thresholds, especially at 15% identity threshold; it achieved 81.13% precision on RV11, outperforming the second best aligner (Dialign) by a big gap of 52.36%. It shows that SymAlign has a much lower tendency than other aligners to over-estimate similarity between structurally non-similar sequences. On the other hand, SymAlign's recalls at the three identity thresholds are very close to those of TM-Align, showing SymAlign's alignments are very close to structure based alignments by TM-align to reflect true structural similarity. As a result, the identity estimated on SymAlign alignments is more informative for structure similarity.

Tables 3 and 4 also show that the recall for TM-align and SymAlign are very close. We estimated the agreement between the two methods (Table 5) and found it to be very strong. This result is especially interesting, considering that TM-align is a structure-based method while SymAlign is merely based on sequence comparisons. SymAlign demonstrates a significant improvement in precision over all other tools in distinguishing between structural similarity and non-similarity based on sequence similarity. Most methods over-estimate sequence identity when fed with a pair of structurally non-similar sequences. Their precisions are close to the ratios of positives on the two datasets.

**Table 5:** The proportions of positive cases both identified by TM-align and SymAlign to those only identified by TM-align with respect to different thresholds.

	SequenceIdentity >10%	SequenceIdentity >15%	Sequence Identity >20%
BAliBASE's RV11	91.76%	89.47%	95.65%
PREFAB	86.39%	90.95%	92.73%

The experiment shows that the agreement between SymAlign and TM-align on RV11 and PREFAB datasets is very strong.

### Sequence alignment visualization

**Figure 3:**
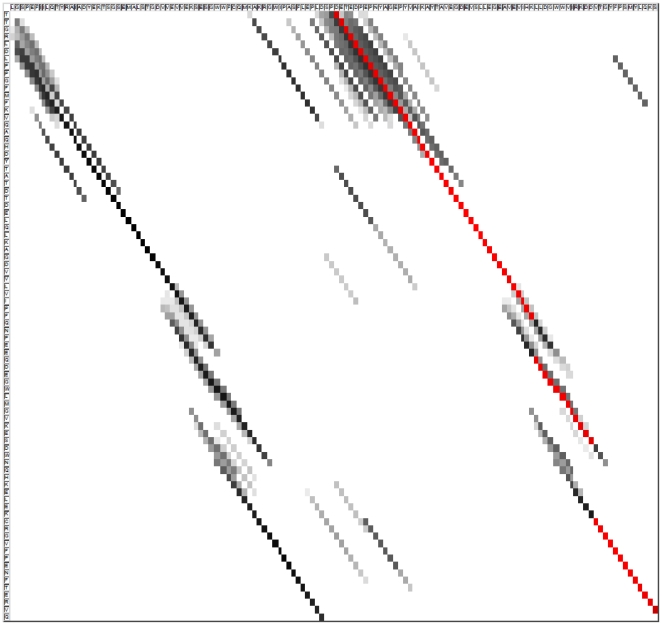
The dot matrix generated by SymAlign for proteins BB11002.1bb9 and BB11002.1ov3_A in RV11. A grayscaled dot represents the number of shared synonyms corresponding to a residue pair. We turn a grayscaled dot into a red-scaled one if the corresponding residue pair is annotated as an equivalent pair in the reference alignment. As one can see, the left side of the matrix shows an alternative alignment with a pattern very similar to the reference alignment.

Most sequence alignment tools only report for each pair of proteins a single alignment with the highest score and provide visualization in the text of sequences to show the alignment result. However, the alignment is not necessarily the best one. Dot matrices are convenient ways to represent alternative alignments. We show here (Figure 3) how the SymAlign can be used to represent the suboptimal alignments between two sequences. The figure shows the alignment of *1bb9* and *1ov3_A* selected from BAliBASE's RV11, where 1bb9 corresponds to the vertical axis while 1ov3_A corresponds to the horizontal axis. Dots are shaded according to a grayscale reflecting the number of shared synonyms associated with each residue pair. The darker the dot, the larger the number of shared synonyms. The reference alignment is illustrated by red dots. As one can see, the left side of the matrix shows an alternative alignment with a pattern very similar to the reference alignment.

We aligned the two sequences according to the two diagonal patterns shown in the figure and measured their RMSDs. The reference alignment produces an RMSD of 9.12 Å, while the other one produces an RMSD of 13.65 Å. This observation is totally consistent with an inspection of the structures showing that 1ov3_A is probably a tetramer and 1bb9 a dimer of some common homologous ancestral repeat.

### Conclusions

In this paper, we present a new method, SymAlign, to align protein sequences, using the concept of protein synonyms, instead of a substitution matrix or a position-specific scoring matrix used by traditional alignment tools to calculate the alignment score between residue pairs. We demonstrate that the shared synonyms can improve the similarity estimate between equivalent residues. SymAlign is evaluated on the most difficult test sets of BAliBASE and PREFAB, and experiments show that SymAlign can align sequences more accurately than alternative methods.

An interesting novelty in the benchmark described here is our assessment of the impact of unrelated sequences. We define a fairly realistic situation to compare various methods. By altering each test set through the addition of unrelated sequences, we demonstrate that SymAlign is very robust to outliers. This should be an essential feature for any sequence aligner because the inclusion of outliers within a group of homologues frequently occurs in sequence analysis.

SymAlign can align sequences to better indicate their preserved 3D structures than standard sequence aligners. On the benchmark datasets, we show that whenever SymAlign delivers an alignment with more than 15% identity, the considered sequences are more likely to be in the same fold.

Furthermore, SymAlign displays not only the optimal but also the sub-optimal alignments on dot-matrices. A fine grayscale makes it easy to identify alternative alignments that may not have been identified by dynamic programming. It is especially useful when aligning distantly related sequences. Altogether SymAlign should prove an interesting development for T-Coffee. The increased accuracy provided by SymAlign will be especially important and useful in all situations where accurately aligning distantly relates homologues is a limiting step.
